# Non-Motor Symptoms and Associated Factors in Parkinson's Disease Patients in Addis Ababa, Ethiopia: A Multicenter Cross-Sectional Study

**DOI:** 10.4314/ejhs.v31i4.19

**Published:** 2021-07

**Authors:** Biniyam A Ayele, Yared Zenebe Zewde, Abenet Tafesse, Amir Sultan, Joseph H Friedman, James H Bower

**Affiliations:** 1 Department of Neurology, College of Health Sciences, Addis Ababa University, Ethiopia; 2 Gastroenterology and Hepatology Division, Department of Internal Medicine, College of Health Sciences, Addis Ababa University; 3 Stanley Aronson Chair in Neurodegenerative Disorders, Director of Movement Disorders Program, Butler Hospital, Department of Neurology, Alpert Medical School of Brown University, Providence, RI, USA; 4 Chair of Division of Movement Disorders, Department of Neurology, Mayo clinic, Rochester, Minnesota, USA

**Keywords:** Non-motor symptoms, Parkinson's disease, young onset PD, Ethiopia

## Abstract

**Background:**

Non-motor symptoms (NMSs) of Parkinson's disease (PD) were often overlooked and less studied. Little is known about NMSs in Ethiopia. The aim of the study was to determine the prevalence of NMSs and associated factors.

**Methods:**

A multi-center cross-sectional observational study was conducted. NMS questionnaire was used to screen for the NMSs. Both descriptive and analytical statistics were used to analyze the data.

**Results:**

Total of 123 PD patients with median of 4 years were investigated. The mean age of PD patients was 62.9 years. The mean age of PD onset was 58.3 years. In 23.6% the age of onset was below age 50. Males accounted 72.4%. Majority of the patients were on Levodopa alone and 31.7% were on levodopa plus trihexyphenidyl. Longer duration of illness was associated with frequent occurrence of NMSs. Constipation was the commonest NMS (78%), followed by urinary urgency (67.5%) and nocturia (63.4%). An unexplained pain was reported by 45.5 %, cognitive impairment (45.5%), and sleep disturbance was reported by 45.5% of the study participants. Neurophysciatric symptoms were reported by small proportion of the patients. Lower monthly earning was associated with swallowing problem, unexplained weight change, and lighheadness.

**Conclusion:**

The prevalence of NMS was high among PD patients in Ethiopia. Constipation was the commonest NMS. Longer duration of illness was associated with frequent occurrence of NMSs. Lower monthly earning was associated with swallowing problem, unexplained weight change, and lighheadness.

## Introduction

Parkinson's disease (PD) is the second commonest neurodegenerative disorder and affects males more than females (1). A recent review indicated the prevalence of PD ranging from 7/100,000 in Ethiopia to 67/100,000 in Nigeria (2). The non-motor symptoms (NMS) of PD are often overlooked and rarely assessed during routine PD patient's evaluation. This is because the motor symptoms such as tremor are often visible to the patients and the family alerting the patients to complain frequently; and they may be unaware that NMS are intrinsic to the disease and therefore may not bring them to the attention of their physicians.

Non-motor symptoms include gastrointestinal, genitourinary, autonomic, cardiovascular, dermatologic and neuropsychiatric symptoms (3–7) Non-motor symptoms typically become prominent in the later stages but often predate the onset of motor symptoms. NMS soften progress with time, and can lead to long-term difficulties (8). Gaps in knowledge surrounding NMS propagate a culture of clinical under-recognition and undertreatment, furthering patient and caregiver stress, and impairing quality of life(8).

Increasing age is considered to be a driving force behind the recently observed increase in prevalence and incidence of Parkinson's disease in sub Saharan Africa (SSA) (2). The non-motor symptoms have tendency to manifest years before the motor features appears; and often associated with increased disability and low quality of life in PD patients (9,10). According to recent reports: neuropsychiatric, gastrointestinal symptoms, and cognitive impairments were the commonest NMSs features reported by African PD patients (11–14). Understanding the non-motor phenotypes of patients with PD in Ethiopia will contribute to the ongoing PD-specific clinical care improvement effort in Ethiopia; and also, it will contribute to the global effort, which aims in understanding the phenotypical and genotypic variation observed between PD patients with different ethnicity, especially those with African ancestors.

The objectives of our study were to determine the prevalence of non-motor symptoms and associated factors in PD patients in Addis Ababa.

## Methods

**Study design, area, and duration**: This is an observational cross sectional survey conducted at neurology clinic in Tikur Anbessa Specialized Hospital (TASH), Zewditu Memorial Hospital (ZMH), Yehuleshet Specialty Clinic (YSC), and Bethezata General Hospital (BGH) between September 2020 and October 2020.TASH is the largest and the oldest tertiary level specialized hospital in Ethiopia with catchment area of close to 8 million populations; and hosts the only neurology training program in the country. ZMH is a general hospital and has long standing clinical and academic affiliation with TASH; and have neurology referral clinic and delivering health services to populations living in Addis Ababa and the surrounding cities. YSC and BGH are private health facilities in the vicinity of TASH and have comprehensive neurology care given by certified neurologist and serving populations in Addis Ababa catchment area.

**Non-Motor Symptoms Questionnaires (NMSQ)**: All 123 Parkinson's disease patients were enrolled from the four health facilities fulfilling UK Parkinson Disease Society Brain Bank (UKBB) diagnostic criteria (15). All the patients were evaluated and interviewed by board certified neurologist. A 30 variable non-Motor Symptoms Questionnaires (NMSQ) was used to screen the patients for non-motor symptoms. Formal authorization letter was obtained from International Parkinson and Movement Disorder Society (MDS) to use NMSQ.

**Study variables**: Non-motor symptoms and young age of PD onset are the dependent variables. The independent variables were demographic variables, H&Y stage, duration of illness, comorbid illness, etc.

**Statistical analysis**: Statistical analysis was done using SPSS version 25. Continuous variables were described using mean, standard deviation (SD), median, and interquartile range (IQR); while categorical variables were described using frequency, proportion, and percentile. Association between dependent variable and predictors was done using logistic regression analysis; and results were presented using odds ratio (OR), confidence interval (CI), and p value. P value < 0.05 was considered statistically significant.

**Ethical considerations**: The study received ethical approval from Addis Ababa University College of Health Sciences Institutional Review Board (IRB) (Protocol number: 117/19/Neuro) and conducted according to the Declaration of Helsinki. All questionnaires were coded to maintain maximum confidentiality. All patients gave an informed written consent before the interview, as approved by our IRB.

## Results

**Baseline characteristics of the study participants**: In the present study, we enrolled total of 123 Parkinson's disease patients from four health facilities (2 public and 2 private) located in Addis Ababa, Ethiopia. The mean age of PD was 62.9 (±12.2) years, while the mean age of PD onset was 58.3 (±10.7) years. Men accounted 72%. Twenty-nine (23.6%) PD patients had age of onset (AOO) below 50 years. The median duration of illness was 4 (IQR: 2 – 6) years. More than half of the patients had H-Y stages of 1 or 2 (53.6%). Fifty-four (38.2%) had H & Y stage 3 or 4. Meanwhile, three patients had clinical features of H & Y stage 5. Fifty (40.7%) of study the participants lived on a monthly income below 27 USD. Seventy-four (60.2%) patients were on Levodopa alone, 31.7% were on Trihexyphenidyl in addition to Levodopa, and 5.7% patients were not yet started on any antiparkinsonian medications (Table 1).

**Table 1:** Baseline characteristics of Parkinson disease patients in Ethiopia (n=123)

Variables	Values
Age in years (mean, 1SD)	62.9 (10.4)
Male (n, %)	89 (72.4)
Female (n, %)	34 (27.6)
Age of PD onset in years (mean, 1SD)	58.3 (10.7)
Duration of illness in years (median, IQR)	4.0 (2 – 6)
NMS score (mean, 1SD)	10.1 (5.9)
H & Y stage (mean, 1SD)	2.2 (1.1)
Monthly income in USD (n, %)	
Below 27 USD	50 (40.7)
Above 27 USD	73 (59.3)
H & Y stage (n, %)	
Stage 1	32 (26)
Stage 2	34 (27.6)
Stage 3	44 (35.8)
Stage 4	10 (2.4)
Stage 5	3 (2.4)
Antiparkinsonian medications (n, %)	
Levodopa alone	74 (60.2)
Trihexyphenidyl + Levodopa	39 (31.7)
Amantadine + Levodopa	2 (1.6)
Dopamine agonist + Levodopa	1 (0.8)
Not yet on medications	7 (5.7)

¶ H & Y: Hoehn and Yahr, USD: US Dollar, SD: Standard deviation, NMS: Non-motor symptoms

**Figure 1:**
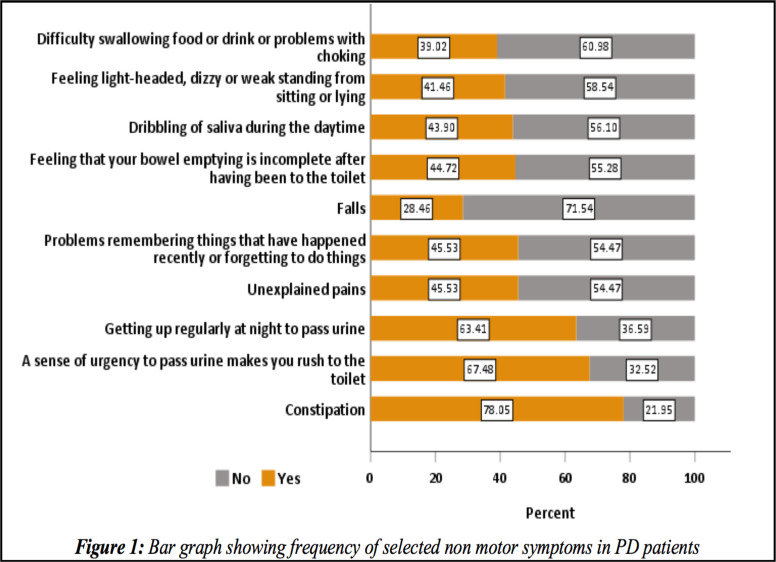
Bar graph showing frequency of selected non motor symptoms in PD patients

**Figure 2:**
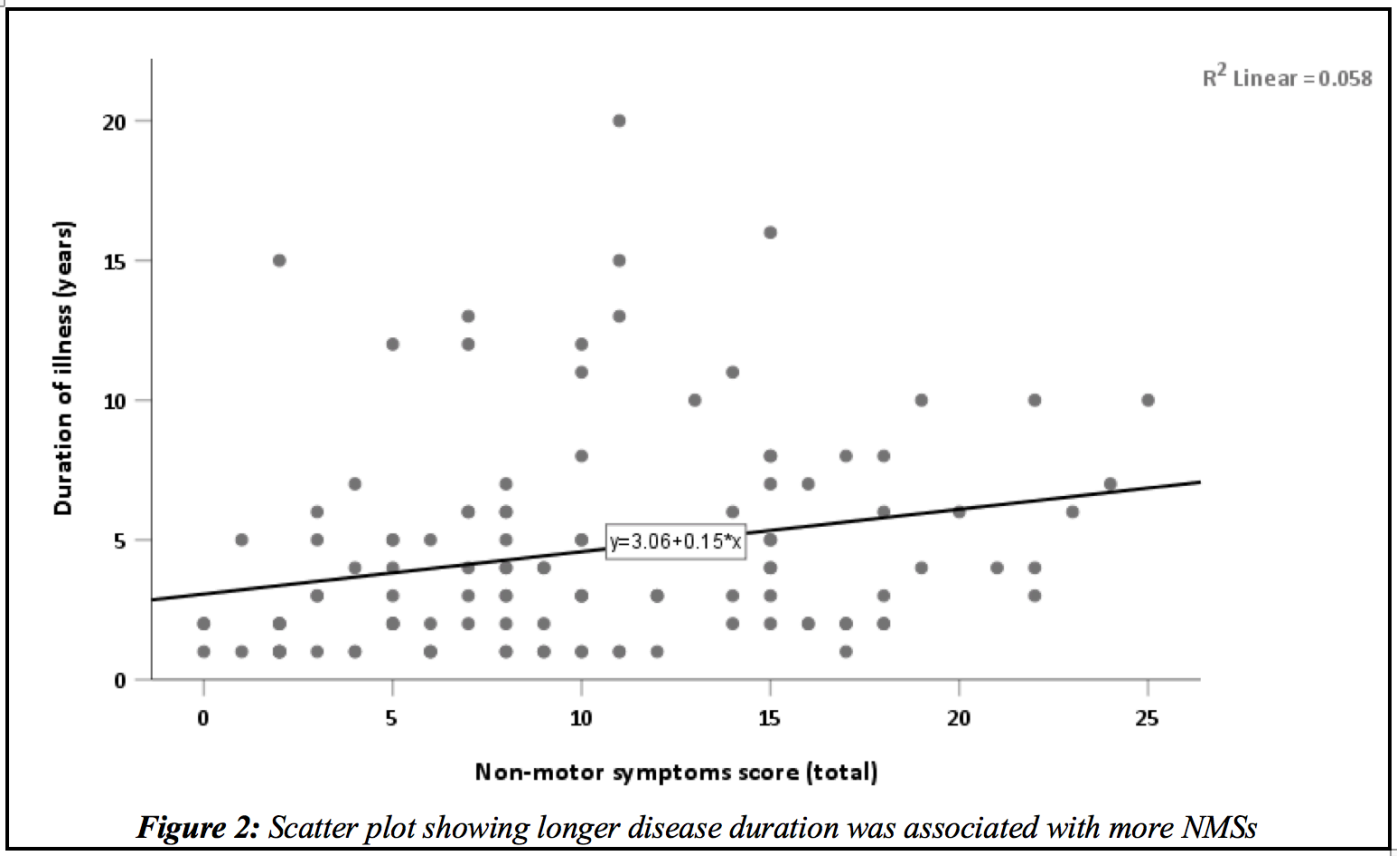
Scatter plot showing longer disease duration was associated with more NMSs

**Figure 3:**
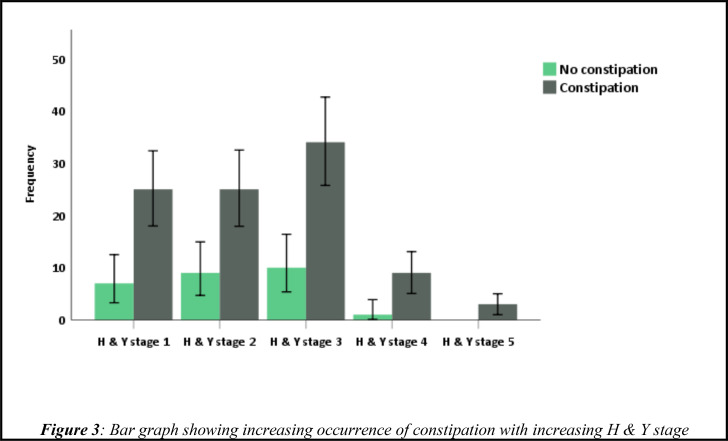
Bar graph showing increasing occurrence of constipation with increasing H & Y stage

**Non-motor symptoms of PD patients**: We used the Non-motor symptoms questionnaire (NMSQ), to screen our patients for NMSs. The NMSQ contains 30 questions and the maximum score is 30 and minimum score is zero. The mean NMSs score was 10.1 (±5.9). A positive correlation was observed between longer disease duration and increased frequency of NMSs (r=0.24, R^2^=0.058, p=0.007) (Figure 2). Three (2.4%) PD patients had a total score of 0. The commonest non-motor symptom was constipation (n=96, 78%). Constipation was reported more by those patients with age of PD onset 60 and above compared to those below 60 (p=0.02). Even though statistically not significant the trend of constipation increased with increasing H & Y stages (p=0.72) (Figure 3). Among total of 39 patients on Trihexyphenidyl, 79.5% reported constipation. Urinary urgency and nocturia were reported by 67.5% and 63.4%, respectively. No difference was observed among young onset PD (AOO<50 years) and PD onset after age 50 regarding urinary urgency (p=0.79) and nocturia (p=0.14). Unexplained pains, falls, forgetfulness, incomplete bowel empting, excessive salivation, and difficulty of getting sleep at night were reported by 45.5 %, 28.5%, 45.5%, 44.7%, 43.9%, and 45.5% respectively (Table 2 and Figure 1). Anosmia/ or ageusia were reported by 25.2% of study participants. Anosmia and ageusia were reported more by those age 50 and above compared to those below age 50 (15.4% vs. 9.8%, p=0.03) (Table 2). Neuropsychiatric symptoms such as: loss of interest in surroundings, feeling sad or blue, feeling anxious, hallucinations, and difficulty of concentrating were reported by 27.6%, 29.3%, 26.8%, 17.1%, and 33.3% respectively. Faecal incontinence was reported only by 8.1% (Table 2).

**Table 2:** Distribution of non-motor symptoms between age of onset (AOO) below and above 50 years

Variables	Total n=123(%)	AOO < 50 n=29, 23.6%	AOO ≥ 50 n=94, 76.4%	p value
Dribbling of saliva during the daytime	54 (43.9)	16 (13)	38 (30.9)	0.2
Loss or change in your ability to taste or smell	31 (25.2)	12 (9.8)	19 (15.4)	0.03
Difficulty swallowing food or drink or problems with choking	48 (39)	11 (8.9)	37 (30.1)	0.89
Vomiting or feelings of sickness (nausea)	17 (13.8)	4 (3.3)	13 (10.6)	0.99
Constipation	96 (78)	21 (17.1)	75 (61)	0.44
Faecal incontinence	10 (8.1)	1 (0.8)	9 (7.3)	0.29
Feeling that your bowel emptying is incomplete after having been to the toilet	55 (44.7)	10 (8.1)	45 (36.6)	0.2
A sense of urgency to pass urine makes you rush to the toilet	83 (67.5)	19 (15.4)	64 (52)	0.79
Getting up regularly at night to pass urine	78 (63.4)	15 (12.2)	63 (51.2)	0.14
Unexplained pains	56 (45.5)	17 (13.8)	39 (31.7)	0.11
Unexplained change in weight	39 (31.7)	9 (7.3)	30 (24.4)	0.93
Problems remembering things that have happened recently or forgetting to do things	56 (45.5)	12 (9.8)	44 (35.8)	0.61
Loss of interest in what is happening around you or in doing things	34 (27.6)	7 (5.7)	27 (22.0)	0.63
Seeing or hearing things that you know or are told are not there	21 (17.1)	2 (1.6)	19 (15.4)	0.09
Difficulty concentrating or staying focused	41 (33.3)	6 (4.9)	35 (28.5)	0.09
Feeling sad, “low” or “blue”	36 (29.3)	9 (7.3)	27 (22.0)	0.81
Feeling anxious, frightened or panicky	33 (26.8)	6 (4.9)	27 (22.0)	0.39
Feeling less interested in sex or more interested in sex	43 (35.0)	10 (8.1)	33 (26.8)	0.95
Finding it difficult to have sex when you try	34 (27.6)	8 (6.5)	26 (21.1)	0.99
Feeling light-headed, dizzy or weak standing from sitting or lying.	51 (41.5)	14 (11.4)	37 (30.1)	0.39
Falling	35 (28.5)	8 (6.5)	27 (22.0)	0.91
Finding it difficult to stay awake during activities such as working, driving or eating.	33 (26.6)	7 (5.7)	26 (21.1)	0.71
Difficulty getting to sleep at night or staying asleep at night	56 (45.5)	14 (11.4)	42 (34.1)	0.73
Intense, vivid or frightening dreams.	47 (38.2)	10 (8.1)	37 (30.1)	0.64
Talking or moving about in your sleep, as if you are “acting out” a dream.	36 (29.3)	9 (7.3)	27 (22.0)	0.81
Unpleasant sensations in your legs at night or while resting, and a feeling that you need to move.	38 (30.9)	7 (5.7)	31 (25.2)	0.37
Swelling of the legs.	17 (13.8)	7 (5.7)	10 (8.1)	0.07
Excessive sweating.	33 (26.8)	10 (8.1)	23 (18.7)	0.29
Double vision	17 (13.8)	7 (5.7)	10 (8.1)	0.07
Believing things are happening to you that other people say are not.	11 (8.9)	1 (0.8)	10 (8.1)	0.24

**Association between NMSs and young onset PD**: Anosmia/or ageusia was associated with late onset PD compared to young onset PD in PD patients in Addis Ababa, Ethiopia; in univariate analysis (OR 0.36, 95% CI 0.11 – 0.88, p=0.02) and when adjusted for other covariates (AOR 0.30, 95% CI 0.10 – 0.92, p=0.04). No significant difference was observed between young onset PD and monthly income, nocturia, unexplained pain, and hallucination. When adjusted for other covariates having double vision showed near-significant association with late onset PD compared to young onset PD (AOR 0.28, 95% CI 0.10 – 1.02, p=0.05) (Table 3).

**Table 3:** Univariate and multivariate analysis of young onset PD covariates

Variables		Univariate analysis			Multivariate analysis		
		
		COR	95% CI	p value	AOR	95% CI	p value
Monthly income							
	Above 27 USD	1			1		
	Below 27 USD	0.86	0.37 – 2.03	0.7	0.67	0.35 – 1.79	0.43
Loss or change in your ability to taste or smell							
	No	1			1		
	Yes	0.36	0.15 – 0.88	0.02	0.30	0.10 – 0.92	0.04
Getting up regularly at night to pass urine							
	No	1			1		
	Yes	1.89	0.81 – 4.42	0.14	2.37	0.89 – 6.33	0.08
Unexplained pains							
	No	1			1		
	Yes	0.50	0.22 – 1.17	0.12	0.36	0.13 – 0.96	0.36
Seeing or hearing things that you know or are told are not there							
	No	1			1		
	Yes	3.42	0.75 – 15.6	0.11	6.80	0.78–59.76	0.08
Difficulty concentrating or staying focused							
	No	1			1		
	Yes	2.27	0.84 – 6.13	0.10	2.57	0.73 – 9.09	0.14
Swelling of the legs							
	No	1			1		
	Yes	0.37	0.13 – 1.09	0.07	0.33	0.08 – 1.37	0.13
Double vision							
	No	1			1		
	Yes	0.37	0.13 – 1.10	0.07	0.28	0.10 – 1.02	0.05

**Association between non-motor symptoms and monthly earning**: Out of the total 123 PD patients in our cohort 40.7% were living on monthly income below 27 USD. Difficulty swallowing food or drink and choking, unexplained change in weight, and feeling light-headed/or dizziness were associated with lower monthly earning (p=0.03, p=0.01, and p=0.02, respectively).

## Discussion

To our best knowledge, this is the first study to assess NMSs among Ethiopian PD patients in Addis Ababa and its surrounding cities. In the present study, the average age of PD patients and age of PD onset were in 6^th^ and 5^th^ decades respectively. Quarter of the patients had young onset PD (AOO<50 years). Males accounted for two-thirds. More than half of the participants had H & Y stage 1 and 2. Constipation was the commonest NMS, followed by urinary urgency and nocturia. Longer disease duration was associated with frequent occurrence of NMSs. Advanced disease stages were associated with increased frequency of NMS.A lower monthly earning was associated with swallowing problem, weight changes, and lightheadness.

In the present study, males accounted for the majority of the participants. Globally, Parkinson's disease is common among elderly males (1). Similar results were reported from the SSA including Ethiopia(13,16,17). In the present study, the age of PD onset was lower compared to the developed countries (18). However, the results were in line with regional report(12). This finding may indicate younger onset PD in African population. Such discrepancy could be related to genetic variation among different ethnicity. The median duration of illness for our patients was four years. This is significantly lower than reports from the western countries (19,20). However, the present results were consistent with previous regional reports (13,18,19). Such short median duration of illness could be due to relative improvement in health care and access to trained neurologist in recent years in most of African countries including Ethiopia. Because of this, diagnosing PD was difficult for long period of time; however, due to recent improvement in neurological care in Ethiopia, more PD patients have been diagnosed.

The majority of the participants had H & Y stage 1 to 3; indicating mild to moderate disease stage in PD patients in Addis Ababa. This results were consistent with global and regional reports(11,12). These results highlights the burden of disease most of our PD patients were suffering from; and further support the need to optimize our care to diagnose and manage PD much earlier. We have observed, increased disease duration and advanced disease stages were associated with increased frequency of non-motor symptoms. These findings are in congruent with the global understanding that, frequency and severity of NMSs increase as the disease duration increase (2,3,20,20). A four years longitudinal observation by Erro R. et al 2016 shows, majority of NMSs increased over time and significantly affected quality of life (21). Such results further strengthen the need to routinely screen our PD patients for NMSs and to manage it early.

In the present study, constipation was reported by majority of the patients. This result is consistent with global and regional reports (5,18). This finding should alert clinicians caring for PD patients in Ethiopia to screen their patients for gastrointestinal symptoms such as constipation regularly and mange accordingly. Likewise, the high prevalence of constipation could be partly attributed to the anticholinergic medications PD patients were prescribed. A significant proportion of the study participants reported urinary urgency and nocturia. These findings were consistent with recent report from Greece, where urinary urgency and nocturia were the commonest non-motor symptoms among 166 PD patients included in their study (9). Such urinary symptoms in elderly patients may be related to symptoms of prostatism or an indicator of faster disease progression among our patients (22–24).

In the present survey, sensory symptoms such as pain and sleep disorders are reported by small proportion of study participants. The current result was consistent with reports from the developed countries (28). Nevertheless, the current result was much lower than the previous reports from Ethiopia, which reported pain in 80% of the study participants (21) and high prevalence of sleep disorders among Ethiopian PD patients(16). These discrepancies are likely due to variations in study methodology and utilization of specific validated tools. In this study, neuropsychiatric symptoms were reported in one-third of the patients. These results are in line with a report from Belgium (10). However, the present results were lower compared to previous reports from the region (13,26). Such differences may be attributable to the variation in study methodology and assessment tools.

Nearly half of the study participant had some degree of cognitive impairment. This finding was in congruent with previous reports from the western countries (31–33). Similarly, the results are similar to reports from the region (12,14,27). This finding warrants the need to screen PD patients in Ethiopia for cognitive impairment using a validated tool. Likewise, we could speculate that the cognitive impairment observed in the current patients may be partly due to chronic use of anticholinergic medications among most of PD patients in Ethiopia. In the present study, faecal incontinence was reported by a small proportion of our patients. This finding was consistent with global figure (35). Similarly, the result was comparable to regional report by Owolabi et al. 2014 (18), who reported faecal incontinence in 6.2% of the participants. This is likely due to small proportion of advanced PD patients in the present cohort, which often associated with more severe NMS such as fecal incontinence.

In the present observation, swallowing problem, unexplained weight changes, and feeling of lighheadness were associated with lower earning. These findings were in line with a population-based study report from Sweden, where low socioeconomic status among PD patients was associated with high mortality(36). Even though there are no available regional and local data to compare these results, we can speculate that, low socioeconomic status could result in malnutrition which will ultimately results in weight loss; similarly, lower monthly incomes could result in inadequate levo-dopa treatment, which will ultimately resulted in manifestation of more severe forms of NMSs such as dysphagia and autonomic dysfunction. A limitation of our study includes; small sample size, lack of sampling design, lack of a control group, and utilization of nonvalidated NMS screening tool.

In conclusion, the prevalence of non-motor symptoms was high among PD patients in Addis Ababa, Ethiopia. Constipation was the commonest NMS. One quarter of our patients had young onset PD. Longer duration of illness was associated with frequent occurrence of NMSs. Swallowing problem, unexplained weight changes, and feeling of lighheadness were associated with lower earning. We recommend future controlled studies of larger samples using validate NMSs screening tools to confirm our findings.

**Ethics approval and consent to participate**: The study received ethical approval from Addis Ababa University College of Health Sciences Institutional Review Board (IRB) (Protocol number: 117/19/Neuro) and conducted according to the Declaration of Helsinki. All subjects provided an informed written consent.
